# Empowering Patients Living With Chronic Conditions Using Video as an Educational Tool: Scoping Review

**DOI:** 10.2196/26427

**Published:** 2021-07-06

**Authors:** Olga Navarro, Marta Escrivá, Raquel Faubel, Vicente Traver

**Affiliations:** 1 Department of Nursing Catholic University of Valencia Valencia Spain; 2 Institute of Information and Communication Technologies Universitat Politècnica de València Valencia Spain; 3 Department of Physiotherapy Universitat de València Valencia Spain; 4 Unidad Mixta de Reingeniería de Procesos Sociosanitarios IIS La Fe-Universitat Politècnica de València Valencia Spain; 5 Physiotherapy in Motion Multispeciality Research Group, Department of Physiotherapy Universitat de València Valencia Spain

**Keywords:** patients, health education, self-care, video, chronic disease

## Abstract

**Background:**

Video is used daily for various purposes, such as leisure, culture, and even learning. Currently, video is a tool that is available to a large part of the population and is simple to use. This audio-visual format has many advantages such as its low cost, speed of dissemination, and possible interaction between users. For these reasons, it is a tool with high dissemination and educational potential, which could be used in the field of health for learning about and management of chronic diseases by adult patients.

**Objective:**

The following review determines whether the use of health educational videos by adult patients with chronic diseases is effective for their self-management according to the literature.

**Methods:**

An electronic literature search of the PubMed, CINAHL, and MEDLINE (via the EBSCOhost platform) databases up to April 2020 was conducted. The systematic scoping review followed the Preferred Reporting Items for Systematic reviews and Meta-Analysis (PRISMA) methodology.

**Results:**

After reviewing 1427 articles, 12 were selected as the most consistent with the proposed inclusion criteria. After their review, it was found that the studies showed that video is effective as a tool for improving care related to chronic diseases.

**Conclusions:**

Video is effective in improving the care and quality of life for patients with chronic diseases, whether the initiative for using video came from their health care professionals or themselves.

## Introduction

### Overview

The increase in the use of social networks and the need for patients to know more about their disease or that of a loved one and how to manage it properly have led to calls for the health system to be updated and health professionals to offer patients reliable and quality tools. Video has become a powerful teaching and dissemination method in all fields. In the health field, it is also used for educational and empowering purposes for the patient, especially when the patient has a chronic disease.

By supplying educational videos for viewing in the health field, the aim is to help patients improve their quality of life through more rigorous self-care, encouraging their access to the health system and often, making personalized care available if video enables live video conferences with health care professionals. Video is a very useful and effective tool, both for professionals and patients, due to its low cost, speed of dissemination, and ease of access.

### Background

The annual report “The Global State of Digital in 2019” created by Hootsuite and We Are Social [1] positioned the YouTube audio-visual platform as the most used social network in Spanish-speaking countries (Mexico, Colombia, Argentina, and Spain). In Spain, 89% of social network users use YouTube [2]. One of the characteristics of the videosocial networks (eg, YouTube or Vimeo) is that the shared videos can host comments and these are registered publicly; in this way, a question that a person asks publicly can be read by other users who have a similar question. This feature, together with its enormous popularity, makes it a tool worth considering for disseminating health information [3,4].

Numerous studies consider that online video content on health is useful and highly effective for educating patients [5,6], although others point out that this content should be viewed with caution since it could be erroneous or confusing and not provide quality information [7-10]. In fact, the most popular videos or those with the highest number of views may contain low-quality or even inappropriate content.

For this reason, many researchers agree on the need for professionals to lead the creation of quality video content [4], although it could also be helpful for patients to do so [11].

It is important to bear in mind that YouTube or Vimeo have their own algorithm for classifying videos that are published, so that, despite efforts from professionals or institutions to generate quality videos, such videos may not reach the target public if they are not properly disseminated and other approaches are not used [12]. Given the lack of quality or patient-specific content on generalist platforms such as YouTube and Vimeo, the main role of the health professional should be that of content curator or link supplier, helping patients to select quality resources [13,14].

The objective of this scoping review is to determine whether the use of educational videos in the health field for self-management of chronic diseases by adult patients is effective.

## Methods

This study follows the guidelines of the Preferred Reporting Items for Systematic reviews and Meta-Analysis (PRISMA) [15]. The PICO (Patient, Intervention, Comparison, Outcomes) framework was used to answer the question: “Does access to audio-visual tools improve the care of patients and/or their families as a way of empowering them to face up to their pathologies?” as shown in Table 1.

**Table 1:** The PICO (Patient, Intervention, Comparison, Outcomes) framework.

PICO framework	Description	Application to this study
P	Definition of the problem or patient	Adult population with chronic diseases or their adult caregivers
I	Interventions	Using video to obtain information about their own illness or a family member's illness
C	Comparison	The effectiveness of viewing videos to improve the care of the chronic pathology to be treated
O	Outcomes= Results	Management of chronic disease in adult patient after the use of video as an educational tool.

The search for journal articles used for this bibliographic review was carried out in the following databases: CINAHL and MEDLINE (via the EBSCOhost platform) and PubMed. The search terms proposed in the process of searching and selecting articles in both databases and the results obtained, in the form of articles, respectively, are shown in the following paragraphs.

The following keywords (MESH descriptors) were used to search for articles: patients, adult, young adult, family, education, health education, self-care, power (psychological), audio-visual aids, video recording, webcasts, chronic disease, chronic pain, caregivers, nursing models. The keywords in Spanish (DECS descriptors) were the following: Pacientes, paciente crónico, familia, cuidadores, educación, autocuidado, vídeo, enfermería, audiovisual, tratamiento, empoderamiento.

To perform this bibliographic review, we selected articles using the following inclusion criteria: articles published between the years 2017 and 2020 (both included); articles dealing with chronic diseases, adult patients, or caregivers; articles where audio-visual content is the main educational tool; studies carried out in humans; text in English and Spanish. All types of sources (academic publications, book publications, reports, and dissertations) included in these databases were accepted. Concerning exclusion criteria, articles on acute diseases or those conducted with pediatric patients were excluded.

**Figure 1:**
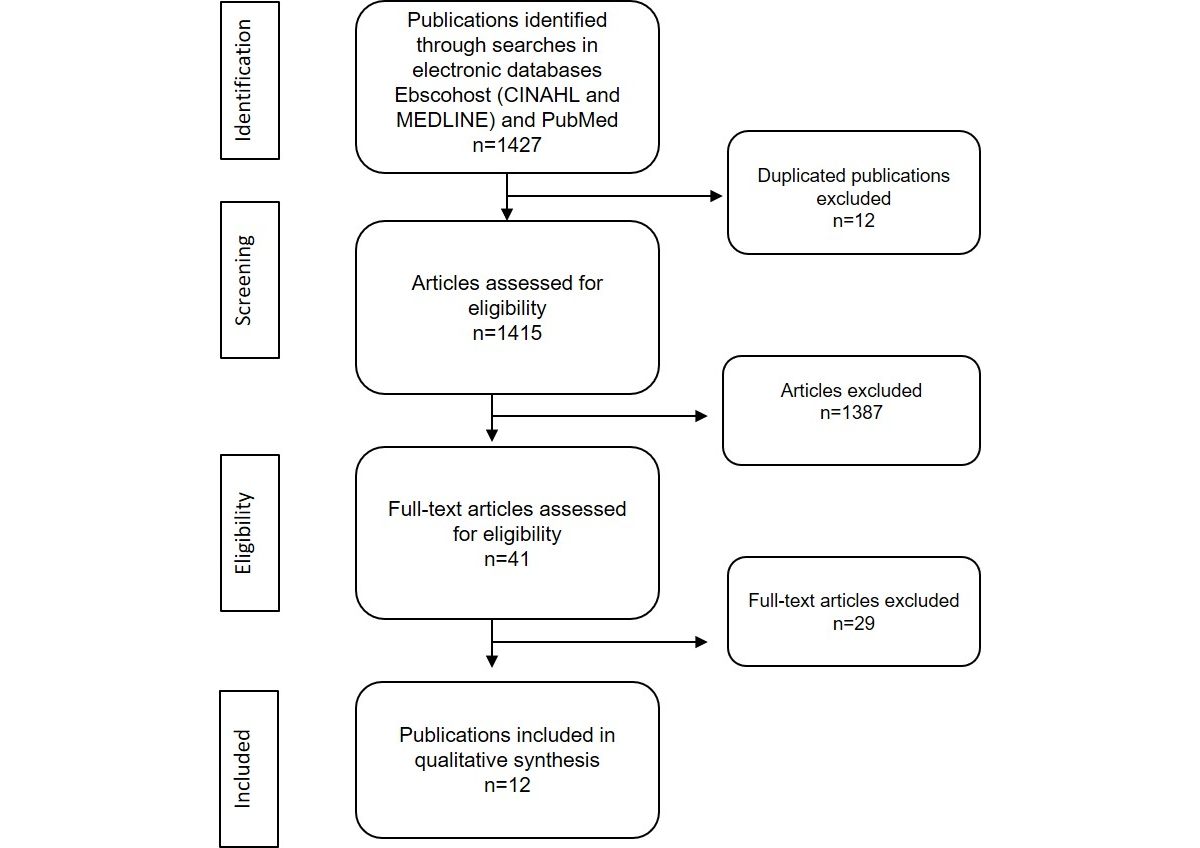
Results of the study selection process using the Preferred Reporting Items for Systematic reviews and Meta-Analysis (PRISMA) methodology.

As shown in the PRISMA flow diagram (Figure 1), after the initial search and eliminating duplicates, 1415 articles were identified, of which 1387 were eliminated after reading the title and summary. Of the 41 remaining, after critical reading of the complete text, 29 other articles were rejected, and 12 studies were finally selected for inclusion in the scoping review.

Methodological quality was assessed using the Downs and Black Checklist for Quality Assessment [16] in the modified version used in several previous studies [17-19]. This checklist is made up of a total of 27 questions classified in 4 domains (reporting, external validity, internal validity, and power). Scores on the modified Downs and Black checklist were classified as “excellent” (score 24-28), “good” (19-23), “fair” (14-18), or “poor” (≤13).

Relevant data regarding study design, characteristics of participants including chronic disease, and interventions carried out including characteristics of the video used as an educational tool were extracted. Specifically, regarding video characteristics, information was collected about the source of the video (created ad hoc for the study or already existing videos) and how it was used as an educational tool (self-administered, shown during an outpatient visit, or shown during a video conference in a synchronous form). Information about results was also extracted related to the expected results previously described in each study. Positive results were considered when a significant improvement in the objectives proposed for the study for the educational tool was shown. 

## Results

In the 12 studies selected to carried out this study, a total of 1398 participants were included. The sample size of the different studies ranged from 8 participants [20] to 429 participants [21], with an average size of 116.5 participants per study. Of the 12 studies, 8 studies included more than 50 subjects. Table 2 summarizes the most relevant data from the articles included.

**Table 2:** Descriptive characteristics of the included studies.

Author(s), year	Title or objective	Study design	Participants	Intervention	Results	Downs and Black Checklist score
Albert et al, 2017 [22]	Factors associated with telemonitoring use among patients with chronic heart failure	Descriptive cross- sectional study	206 outpatients and hospitalized patients with heart failure	A 6-minute video was offered that dealt with telemonitoring, which was intended to collect information on the patient's blood pressure and weight to send to the caregiver. Subsequently, questionnaires were used to evaluate the efficacy.	The intervention was well received by the patients, especially when satisfying the personal needs of care and learning in a bidirectional way with a doctor or a nurse, through the use of smartphones.	16
Rosen et al, 2017 [23]	Telehealth protocol to prevent readmission among high-risk patients with congestive heart failure	Quasiexperimental study	50 patients with congestive heart failure	A telehealth platform was developed that enables, through videoconferencing, educating patients about their disease, to prevent hospital readmissions for congestive heart failure.	Adherence to treatment was increased, and there was a marked decrease in readmission of patients with congestive heart failure.	19
Vogler et al, 2017 [24]	Assessing outcomes of educational videos in group visits for patients with chronic pain at an academic primary care clinic	Analytical observational prospective cohort study	14 patients with chronic noncancerous pain who underwent an educational program on pain	Educational videos were offered to patients and discussed orally in a subsequent group visit. In total, there were 4 group visits.	The study participants, by improving their knowledge, reduced the doses of painkillers they took, in addition to reducing visits to the emergency room for pain.	17
Farver-Vestergaard et al, 2019 [20]	Teledelivered mindfulness-based cognitive therapy in chronic obstructive pulmonary disease: a mixed methods feasibility study	Quasiexperimental study	8 patients with COPD^a^ underwent Mindfulness-Based Cognitive Therapy through videoconferencing	Two groups underwent mindfulness therapy to reduce psychological distress and improve the physical health of COPD through video conference sessions.	Clinical improvement was observed in hospital depression and anxiety. The patients perceived an improvement in unpleasant physical sensations and psychological symptoms.	18
Ward et al, 2018 [25]	Evaluation of multidisciplinary pulmonary rehabilitation education delivered by either DVD or spoken talk	Analytical observational cohort study	123 patients with COPD in a pulmonary rehabilitation study	Two groups were divided: One was provided with education about pulmonary rehabilitation through DVD, and the other group received the same information through oral and face-to-face discussion.	Education via DVD was found to be as effective as traditional education.	16
Ketelaars et al, 2017 [26]	The effect of video information on anxiety levels in women attending colposcopy: a randomized controlled trial	Randomized controlled clinical trial	136 women, older than 18 years, with a positive hrHPV^b^ test, referred for colposcopy	Group A received information about colposcopy through a video and a brochure. Group B was only given the brochure.	The video did not significantly reduce the levels of anxiety, depression, or pain in the participants. But participants positively valued the video information.	17
De Lepeleere et al, 2017 [27]	The effect of an online video intervention “movie models” on specific parenting practices and parental self-efficacy related to children’s physical activity, screen-time and healthy diet	Quasiexperimental two-arm study	238 parents with children 6-12 years old	The study offered 22 online, 2-minute videos on obesity and chronicity prevention for 4 weeks.	Parents valued the video as a useful and applicable tool. It was an effective tool for improving family habits and parental self-efficacy.	20
Bakas et al, 2019 [28]	Using telehealth to optimize healthy independent living for older adults: a feasibility study	Quasiexperimental study	22 older adults with some chronic health condition	A textbook, advice sheets, and 2 DVDs were provided; 3 telepresence sessions were held where the patients were trained using the tools provided.	Improvements were found in quality of life, self-efficacy, and confidence perceived by the patients.	17
Zanaboni et al, 2017 [29]	Long-term exercise maintenance in COPD via telerehabilitation: a 2-year pilot study	Quasiexperimental study	10 adult COPD patients	The intervention consisted of providing patients with exercises at home, supervised by videoconference by a physiotherapist.	It was determined that telerehabilitation is feasible for maintaining good long-term health status in COPD patients.	20
Taylor et al, 2018 [30]	Integrating innovative telehealth solutions into an interprofessional team-delivered chronic care management pilot program	Retrospective observational study	69 patients with 3 or more chronic pathologies, taking at least 5 drugs	A teleconsultation service was provided with a pharmacist to review treatments, doses, and improve adherence.	One-third of the patients changed their habits after pharmaceutical advice. It was found to be a useful tool for reducing errors.	15
McLeod et al, 2020 [21]	Impact of a comprehensive digital health programme on HbA1c and weight after 12 months for people with diabetes and prediabetes: a randomised controlled trial	Randomized controlled trial	429 patients with diabetes not taking insulin and daily access to the internet	The control and intervention arms received usual care. The intervention arm received the BetaMe/Melon program over 12 months, delivered through mobile devices and web-based platforms.	There were small improvements in HbA1c^c^ and weight at 4 months that had largely attenuated by 12 months. The BetaMe/ Melon program in its current form cannot be recommended for use in the management of diabetes or prediabetes.	25
Locke et al, 2019 [31]	Using video telehealth to facilitate inhaler training in rural patients with obstructive lung disease	Retrospective observational analytical cohort study	93 resident patients with COPD or asthma in a rural setting	Live video training sessions were given to patients to explain the use of inhalers.	Improvement in inhalation technique was achieved by patients with asthma or COPD.	19

^a^COPD: chronic obstructive pulmonary disease.

^b^hrHPV: high-risk human papillomavirus.

^c^HbA1c: glycosylated hemoglobin.

All videos employed as educational tools were specifically created within the frame of research. None of the studies included in the review used public videos or videos already hosted on a YouTube channel.

Regarding the study design, 5 of the studies were designed as a quasiexperimental, pre-post study. Another 5 studies were observational with either a retrospective, prospective, or descriptive design. In those studies, the objective was to assess the impact of videos as an educational tool provided to a group of patients for training or follow-up. Only 2 of the studies were designed as a randomized controlled trial.

Regarding the chronic disease of the participants, the selected studies included participants with diagnoses of chronic obstructive pulmonary disease (COPD; 4/12, 33%) [20,25,29,31], which was the most frequent; heart failure (2/12, 17%) [22,23]; chronic pain (1/12, 8%) [24]; diabetes (1/12, 8%) [21]; or squamous intraepithelial lesion due to human papillomavirus (HPV; 1/12, 8%) [26]. Participants with any chronic conditions were included in 3 of the studies [27,28,30], while quality of life was measured in 25% (3/12) of the studies.

Each study included in the review provided different educational video tools to the subjects who participated in the research: educational video, informative video, video based on real cases, explanatory video. They were provided in different ways and in some cases combined with other educational strategies. Specifically, some studies used videoconferencing to provide physical therapy exercises [29], mindfulness therapy [20], pharmacological advice [30], or training about using inhalers [31]. Other studies used online videos [26,27] for different purposes such as education for pain management [24], information about diagnostic procedures [26], or disease prevention [27]. One study took advantage of an outpatient visit or hospitalization for the patient to view 1 short video about telemonitoring options for their disease [22]. In other studies, videos were provided in a DVD delivered to the patient independently [25] or combined with other activities such as videoconference sessions [28]. Lastly, some studies included an educational video combined with other tools incorporated in a complex platform for telehealth [21,23].

These studies assessed whether the application of video had improved the management of chronic disease, and successful results were obtained in most cases. Of the 12 studies included in the review, 2 studies [21,26] did not show significant results to support the hypothesis that video is an effective tool to improve the health of patients with chronic disease in the long term. The study carried out by Ketelaars et al [26] found that the educational video did not significantly reduce the anxiety levels of the participants; that is, it was not effective as a stress reduction tool for women with HPV before a diagnostic procedure, compared with a brochure. However, the patients positively valued the videos offered. On the other hand, McLeod et al [21] found positive results on different outcomes in the first few months of a complex intervention for patients with diabetes. Nevertheless, after 12 months of follow-up, there were no significant differences.

Positive significant results were found in 10 of the 12 studies with a great heterogeneity of outcome variables: The doses of drugs and analgesics [24,30], exacerbations of chronic diseases, readmissions, and emergency room visits [23,24] were all reduced. Studies aimed specifically at patients with COPD showed that rehabilitation using video resources is also effective [25,29,31]. In addition to this, studies also found a decrease in pain, anxiety, and depression [20].

Lastly, improvements in disease knowledge [25], satisfaction [27], and health-related quality of life [28] were also found. These data indicate the effectiveness of the audio-visual tools provided to patients for improving management of chronic diseases and reinforcing health advice.

In terms of methodological quality, according to the modified version of the Downs and Black Checklist for Quality Assessment (Multimedia Appendix 1), 7 of the studies presented with a fair quality grade (scores from 15 to 18), and the other 5 studies had excellent or good methodological quality (scores from 19 to 25). The average score was 18.25. Items with the worst ratings on the quality scale were those related to blinding and randomization.

## Discussion

### Principal Findings

This scoping review identified 1427 articles, of which 12 fulfilled the selection criteria to determine the effectiveness of video as an educational and empowering tool for patients with chronic disease and their caregivers. The results of the review showed that audio-visual instruments are highly effective in the acquisition of competencies by the patient for self-management of chronic diseases. According to the studies reviewed, patients and caregivers themselves were able to improve their self-care and management of chronic diseases after complementing health advice with specific video resources.

In all the articles analyzed in this review, professionals offered the patient or group of patients videos or different audio-visual instruments specifically developed for research. Data on videos freely consulted on the internet by the patient were not included in these articles, nor were different video formats (eg, videoconferencing, DVD viewing, informative or explanatory video, educational video) and their effectiveness compared.

The use of educational videos could contribute to improving the general health status of patients with chronic disease and could even act at a preventive level, reducing the number of admissions and hospital stays. Along these lines, the use of educational videos with adult patients with chronic disease could help reduce anxiety, depression, pain, and even rescue medication doses that patients routinely take.

In addition, educational videos could be a powerful tool for the empowerment of patients with chronic diseases, helping to resolve their issues in a fast, economical, and dynamic way.

It was observed that in all the studies reviewed, health professionals directly offered the video or other audio-visual instruments to a group of patients, and patients did not freely search for videos on the internet. This issue omits investigations where a significant proportion of the population consults and shares videos on the internet, and thus, we were unable to affirm whether in this freer area, not guided by a professional, there are differences in effectiveness.

If audio-visual tools were used in the health system, patients could improve their health status and decrease their admissions and stays in hospital. In addition to this, they could resolve issues wherever they may be, in a fast, economical, and dynamic way.

The implementation of audio-visual tools could reduce the workload of health professionals, reach a greater number of people, decrease health spending, and carry out more effective health education, among other possible benefits.

In this review, in most of the studies, the professionals reinforced the videos with other types of supplementary educational material, such as infographics, brochures, and face-to-face visits.

Despite these results, video is not yet implemented in clinical practice as an educational tool of great value, perhaps due to the scarce evidence in this regard and the lack of training for professionals, among other factors.

Health professionals could have a relevant part in developing or selecting useful and high-quality video material for patients, particularly since videos available via services such as YouTube or Vimeo should be screened by an expert before patients view them, to increase the level of safety and trust in the content [32]. Indeed, material obtained in these video platforms without supervision could even contain dangerous information for patients’ health [33,34].

For this reason, it would be useful for health professionals to be updated in the use of new technologies and more specifically video, since audio-visual instruments are highly effective in the management of chronic diseases. Despite this, these tools, in some of the studies that were reviewed, had to be reinforced with additional information that was intelligible by the entire population, including the elderly population.

Although audio-visual tools are effective for improving the management of pathologies, personalized attention should not be omitted when necessary, so that the combined effectiveness of all educational instruments persists over time and is retained in the population. Good communication between patients and health professionals, either face-to-face or using videoconference, will reduce the need to get additional information. That would avoid browsing video material with low scientific evidence that could be harmful for the patient [35].

The present scoping review was conducted following the PRISMA checklist. Despite this, one of the limitations we found was the low number of articles included in the review and the great heterogeneity in the design of the included studies and chronic diseases included. On the other hand, the methodological quality of some of the studies could be explained by the challenge of recruiting participants for educational research studies and designing randomized controlled trials with a long-term follow-up. At the same time, the diverse range of aspects related to chronicity analyzed in the included studies also represents that using video as an educational and empowering tool for patients with chronic disease could improve several aspects of the disease.

With regard to chronic diseases, after reviewing the 12 selected articles, we concluded that the authors attached great importance to a group of chronic diseases such as COPD, heart disease, and diabetes. On the other hand, there are various chronic diseases that have a high impact on the population and were not included in the studies (eg, hypertension, obesity, fibromyalgia, and dementias); therefore, it would be useful to include them in this type of research. Further possible benefits in the use of video might be found.

On the other hand, the authors suggested tools such as videoconferencing with health care professionals (ie, nurses, doctors, pharmacists) and showed that after use of such tools, significant improvements in patient education are obtained. However, these types of resources are not widely used in health services either. It would be appropriate to study the barriers that hinder the use of these tools when receiving and offering health information through videos, such as lack of expertise in use, lack of technical resources, security, or support problems from the health system.

### Conclusions

Despite the conclusion of the effectiveness of video for educating patients and improving self-care for chronic diseases in different articles published in well-known and high-impact medical journals, the use of video in the health field has not yet been implemented on a routine basis. It would be advisable to continue researching in this area and identify the advantages and benefits that audio-visual instruments can bring to patients with chronic diseases, their caregivers, and health professionals themselves, consolidating video as a complementary tool and of great support in reinforcing the health advice offered in consultations and clinical events.

## Appendix

Multimedia Appendix 1 Methodological quality according to the modified version of Downs and Black Checklist for Quality Assessment.

## Abbreviations

COPD: Chronic obstructive pulmonary disease
HPV: human papillomavirus
PICO: Patient, Intervention, Comparison, Outcomes
PRISMA: Preferred Reporting Items for Systematic reviews and Meta-Analysis

## References

[1] The global state of digital in 2019. Hootsuite.

[2] Newberry C, Adame A (2019). 22 Estadísticas de YouTube esenciales para este año. Hootsuite.

[3] Khatri P, Singh SR, Belani NK, Yeong YL, Lohan R, Lim YW, Teo WZ (2020). YouTube as source of information on 2019 novel coronavirus outbreak: a cross sectional study of English and Mandarin content. Travel Med Infect Dis.

[4] Rubel KE, Alwani MM, Nwosu OI, Bandali EH, Shipchandler TZ, Illing EA, Ting JY (2020). Understandability and actionability of audiovisual patient education materials on sinusitis. Int Forum Allergy Rhinol.

[5] Altan Şallı G, Egil E (2020). Are YouTube videos useful as a source of information for oral care of leukemia patients?. Quintessence Int.

[6] Ruiz-Roca JA, Martínez-Izquierdo A, Mengual-Pujante D, López EPF, López-Jornet P (2020). Is YouTube a useful tool for oral care in patients with Parkinson's disease?. Spec Care Dentist.

[7] Jain N, Abboudi H, Kalic A, Gill F, Al-Hasani H (2019). YouTube as a source of patient information for transrectal ultrasound-guided biopsy of the prostate. Clin Radiol.

[8] Cassidy JT, Fitzgerald E, Cassidy ES, Cleary M, Byrne DP, Devitt BM, Baker JF (2018). YouTube provides poor information regarding anterior cruciate ligament injury and reconstruction. Knee Surg Sports Traumatol Arthrosc.

[9] Leong AY, Sanghera R, Jhajj J, Desai N, Jammu BS, Makowsky MJ (2018). Is YouTube Useful as a Source of Health Information for Adults With Type 2 Diabetes? A South Asian Perspective. Can J Diabetes.

[10] Lambert K, Mullan J, Mansfield K, Koukomous A, Mesiti L (2017). Evaluation of the quality and health literacy demand of online renal diet information. J Hum Nutr Diet.

[11] Salama A, Panoch J, Bandali E, Carroll A, Wiehe S, Downs S, Cain MP, Frankel R, Chan KH (2020). Consulting "Dr. YouTube": an objective evaluation of hypospadias videos on a popular video-sharing website. J Pediatr Urol.

[12] Fernandez-Llatas C, Traver V, Borras-Morell J, Martinez-Millana A, Karlsen R (2017). Are Health Videos from Hospitals, Health Organizations, and Active Users Available to Health Consumers? An Analysis of Diabetes Health Video Ranking in YouTube. Comput Math Methods Med.

[13] Al-Busaidi IS, Anderson TJ, Alamri Y (2017). Qualitative analysis of Parkinson's disease information on social media: the case of YouTube™. EPMA J.

[14] Lee JL, Frey M, Frey P, Hollin IL, Wu AW (2017). Seeing is Engaging: Vlogs as a Tool for Patient Engagement. Patient.

[15] Moher D, Shamseer L, Clarke M, Ghersi D, Liberati A, Petticrew M, Shekelle P, Stewart LA, PRISMA-P Group (2015). Preferred reporting items for systematic review and meta-analysis protocols (PRISMA-P) 2015 statement. Syst Rev.

[16] Downs SH, Black N (1998). The feasibility of creating a checklist for the assessment of the methodological quality both of randomised and non-randomised studies of health care interventions. J Epidemiol Community Health.

[17] Trac MH, McArthur E, Jandoc R, Dixon SN, Nash DM, Hackam DG, Garg AX (2016). Macrolide antibiotics and the risk of ventricular arrhythmia in older adults. CMAJ.

[18] Hooper P, Jutai JW, Strong G, Russell-Minda E (2008). Age-related macular degeneration and low-vision rehabilitation: a systematic review. Can J Ophthalmol.

[19] O'Connor SR, Tully MA, Ryan B, Bradley JM, Baxter GD, McDonough SM (2015). Failure of a numerical quality assessment scale to identify potential risk of bias in a systematic review: a comparison study. BMC Res Notes.

[20] Farver-Vestergaard I, O’Connor M, Smith NC, Løkke A, Bendstrup E, Zachariae R (2018). Tele-delivered mindfulness-based cognitive therapy in chronic obstructive pulmonary disease: A mixed-methods feasibility study. J Telemed Telecare.

[21] McLeod M, Stanley J, Signal V, Stairmand J, Thompson D, Henderson K, Davies C, Krebs J, Dowell A, Grainger R, Sarfati D (2020). Impact of a comprehensive digital health programme on HbA and weight after 12 months for people with diabetes and prediabetes: a randomised controlled trial. Diabetologia.

[22] Albert NM, Dinesen B, Spindler H, Southard J, Bena JF, Catz S, Kim TY, Nielsen G, Tong K, Nesbitt TS (2017). Factors associated with telemonitoring use among patients with chronic heart failure. J Telemed Telecare.

[23] Rosen D, McCall JD, Primack BA (2017). Telehealth Protocol to Prevent Readmission Among High-Risk Patients With Congestive Heart Failure. Am J Med.

[24] Vogler CN, Sattovia S, Salazar LY, Leung TI, Botchway A (2017). Assessing outcomes of educational videos in group visits for patients with chronic pain at an academic primary care clinic. Postgrad Med.

[25] Ward S, Sewell L, Singh S (2018). Evaluation of multidisciplinary pulmonary rehabilitation education delivered by either DVD or spoken talk. Clin Respir J.

[26] Ketelaars PJW, Buskes MHM, Bosgraaf RP, van Hamont D, Prins JB, Massuger LFAG, Melchers WJG, Bekkers RLM (2017). The effect of video information on anxiety levels in women attending colposcopy: a randomized controlled trial. Acta Oncol.

[27] De Lepeleere S, De Bourdeaudhuij I, Cardon G, Verloigne M (2017). The effect of an online video intervention 'Movie Models' on specific parenting practices and parental self-efficacy related to children's physical activity, screen-time and healthy diet: a quasi experimental study. BMC Public Health.

[28] Bakas T, Sampsel D, Israel J, Chamnikar A, Bodnarik B, Clark JG, Ulrich MG, Vanderelst D (2018). Using telehealth to optimize healthy independent living for older adults: A feasibility study. Geriatr Nurs.

[29] Zanaboni P, Hoaas H, Aarøen Lien L, Hjalmarsen A, Wootton R (2016). Long-term exercise maintenance in COPD via telerehabilitation: a two-year pilot study. J Telemed Telecare.

[30] Taylor AM, Bingham J, Schussel K, Axon DR, Dickman DJ, Boesen K, Martin R, Warholak TL (2018). Integrating Innovative Telehealth Solutions into an Interprofessional Team-Delivered Chronic Care Management Pilot Program. J Manag Care Spec Pharm.

[31] Locke ER, Thomas RM, Woo DM, Nguyen EHK, Tamanaha BK, Press VG, Reiber GE, Kaboli PJ, Fan VS (2019). Using Video Telehealth to Facilitate Inhaler Training in Rural Patients with Obstructive Lung Disease. Telemed J E Health.

[32] Gabarron E, Fernandez-Luque L, Armayones M, Lau AY (2013). Identifying Measures Used for Assessing Quality of YouTube Videos with Patient Health Information: A Review of Current Literature. Interact J Med Res.

[33] Mueller SM, Hongler VNS, Jungo P, Cajacob L, Schwegler S, Steveling EH, Manjaly Thomas Z, Fuchs O, Navarini A, Scherer K, Brandt O (2020). Fiction, Falsehoods, and Few Facts: Cross-Sectional Study on the Content-Related Quality of Atopic Eczema-Related Videos on YouTube. J Med Internet Res.

[34] Syed-Abdul S, Fernandez-Luque L, Jian W, Li Y, Crain S, Hsu M, Wang Y, Khandregzen D, Chuluunbaatar E, Nguyen PA, Liou D (2013). Misleading health-related information promoted through video-based social media: anorexia on YouTube. J Med Internet Res.

[35] Langford A, Loeb S (2019). Perceived Patient-Provider Communication Quality and Sociodemographic Factors Associated With Watching Health-Related Videos on YouTube: A Cross-Sectional Analysis. J Med Internet Res.

