# Supplementary material for: Empowering Patients Living With Chronic Conditions Using Video as an Educational Tool: Scoping Review
Source: J Med Internet Res. 2021 Jul 6;23(7):e26427. doi: 10.2196/26427 (PMC8292934; doi:10.2196/26427)
Supplement: Multimedia Appendix 1 [file jmir_v23i7e26427_app1.docx]

Table 1. Methodological quality according to the modified version of Downs and Black Checklist for Quality Assessment

|  | Albert 2017  [15] | Rosen 2017  [16] | Vogler  2017  [17] | Farver-  Vestergaard  2019  [18] | Ward 2018  [19] | Ketelaars  2017  [20] | De Lepeleere 2017  [21] | Bakas 2019  [22] | Zanaboni  2017  [23] | Taylor 2018  [24] | McLeod 2020  [25] | Locke 2019  [26] |
| --- | --- | --- | --- | --- | --- | --- | --- | --- | --- | --- | --- | --- |
| REPORTING |  | | | | | | | | | | | |
| Q1 Hypothesis/aim/objective clearly described | 1 | 1 | 1 | 1 | 1 | 1 | 1 | 1 | 1 | 1 | 1 | 1 |
| Q2 Main outcomes in Introduction or Methods | 1 | 1 | 1 | 1 | 0 | 0 | 1 | 0 | 1 | 0 | 1 | 1 |
| Q3 Patient characteristics clearly described | 1 | 1 | 1 | 1 | 0 | 1 | 1 | 1 | 1 | 1 | 1 | 1 |
| Q4 Interventions of interests clearly described | 1 | 0 | 1 | 1 | 1 | 1 | 1 | 1 | 1 | 1 | 1 | 1 |
| Q5 Principal confounders clearly described | 0 | 0 | 0 | 0 | 0 | 0 | 0 | 0 | 0 | 0 | 1 | 1 |
| Q6 Mainly findings clearly described | 1 | 1 | 1 | 1 | 1 | 1 | 1 | 1 | 1 | 1 | 1 | 1 |
| Q7 Estimates of random variability provided for main outcomes | 1 | 1 | 1 | 1 | 1 | 1 | 1 | 1 | 1 | 1 | 1 | 1 |
| Q8 All adverse events of intervention reported | 1 | 1 | 1 | 1 | 1 | 1 | 1 | 1 | 1 | 1 | 1 | 1 |
| Q9 Characteristics of patients lost to follow-up described | 1 | 1 | 0 | 1 | 0 | 1 | 1 | 1 | 0 | 0 | 1 | 0 |
| Q10 Probability values reported for main outcomes | 1 | 1 | 1 | 1 | 1 | 0 | 0 | 0 | 1 | 0 | 0 | 0 |
| EXTERNAL VALIDITY |  | | | | | | | | | | | |
| Q11 Subjects asked to participate were representative of source population | UTD | UTD | UTD | UTD | UTD | UTD | 1 | UTD | UTD | UTD | 1 | UTD |
| Q12 Subjects prepared to participate were representative of source population | UTD | 1 | UTD | UTD | UTD | UTD | 1 | UTD | UTD | UTD | 1 | UTD |
| Q13 Location and delivery of study was representative of source population | 1 | 1 | 1 | 1 | 1 | 1 | 1 | 1 | 1 | 1 | 1 | 1 |
| INTERNAL VALIDITY- BIAS & CONFOUNDING |  | | | | | | | | | | | |
| Q14 Study participants blinded to treatment | 0 | 0 | 0 | 0 | UTD | 0 | UTD | 0 | 1 | 0 | 1 | 0 |
| Q15 Blinded outcome assessment | 0 | UTD | UTD | UTD | UTD | 0 | 0 | 0 | 1 | 0 | 1 | 0 |
| Q16 Any data dredging clearly described | 1 | 1 | 1 | 1 | 1 | 1 | 1 | 1 | 1 | 1 | 1 | 1 |
| Q17 Analyses adjust for differing lengths of follow-up | 1 | 1 | 1 | 1 | 1 | 1 | 1 | 1 | 1 | 1 | 1 | 1 |
| Q18 Appropriate statistical test performed | 1 | 1 | 1 | 1 | 1 | 1 | 1 | 1 | 1 | 1 | 1 | 1 |
| Q19 Compliance with interventions was reliable | 1 | 1 | 1 | 1 | 1 | 1 | 1 | 1 | 1 | 1 | 1 | 1 |
| Q20 Outcome measures were reliable and valid | 1 | 1 | 1 | 1 | 1 | 1 | 1 | 1 | 1 | 1 | 1 | 1 |
| Q21 All participants recruited from the same source population | 0 | 1 | 1 | 1 | 1 | 1 | 1 | 1 | 1 | 1 | 1 | 1 |
| Q22 All participants recruited over the same time period | 1 | 1 | 1 | 1 | 1 | 1 | 1 | 1 | 1 | 1 | 1 | 1 |
| Q23 Participants randomized to treatment | 0 | 0 | 0 | 0 | UTD | 1 | 1 | 1 | 0 | 0 | 1 | 0 |
| Q24 Allocation of treatment concealed from investigators and participants | 0 | 0 | 0 | 0 | 0 | 0 | 0 | 0 | 0 | 0 | 0 | 0 |
| Q25 Adequate adjustment for confounding | 0 | UTD | 0 | 0 | 0 | 0 | 0 | 0 | 0 | 0 | 1 | 1 |
| Q26 Losses to follow-up taken into account | UTD | 1 | 1 | 1 | 1 | 1 | 1 | 1 | 1 | 0 | 1 | 1 |
| POWER |  | | | | | | | | | | | |
| Q27 Sufficient power to detect treatment significance level of 0.05 | 0 | 1 | 0 | 0 | 1 | 0 | 0 | 0 | 1 | 1 | 1 | 1 |
| **TOTAL** | **16** | **19** | **17** | **18** | **16** | **17** | **20** | **17** | **20** | **15** | **25** | **19** |

Note: UTD: Unable to determine
